# Neuroprotective effects of *Paederia foetida* Linn. on scopolamine-induced cognitive impairment in rats

**DOI:** 10.14202/vetworld.2024.1972-1982

**Published:** 2024-09-01

**Authors:** Narawut Pakaprot, Tanaporn Khamphaya, Pattamaporn Kwankaew, Sarawut Ninsuwan, Sutida Laisunthad, Kotchaporn Thonoi, Saruda Kuraeiad

**Affiliations:** 1 Department of Physiology, Faculty of Medicine Siriraj Hospital, Mahidol University, Srisavarindhira Bldg., 13^th^ Floor, Wanglang Road, Siriraj Subdistrict, Bangkoknoi District, Bangkok, 10700, Thailand; 2 Department of Occupational Health and Safety, School of Public Health, Walailak University, Nakhon Si Thammarat, 80160, Thailand; 3 Department of Medical Technology, School of Allied Health Sciences, Walailak University, Nakhon Si Thammarat, 80160, Thailand; 4 Research Center in Tropical Pathobiology, Walailak University, Nakhon Si Thammarat, 80160, Thailand

**Keywords:** Alzheimer’s disease, neuroprotection, oxidative stress, *Paederia foetida*, scopolamine

## Abstract

**Background and Aim:**

Alzheimer’s disease (AD) poses a significant health-care challenge, often linked to cognitive decline caused by oxidative stress. This study investigated the potential neuroprotective effects of the *Paederia foetid*a leaf extract (PFE) in rats that exhibited scopolamine-induced dementia mimicking AD.

**Materials and Methods:**

Forty-two male rats were treated with either donepezil (0.5 mg/kg) or PFE at doses of 250, 500, and 1000 mg/kg for 14 days before and 14 days after the beginning of Alzheimer’s-like symptoms after 14 consecutive days of scopolamine administration. Behavioral tests, including the open-field test for locomotor activity and the Morris water maze task for learning and memory assessment, were conducted. Neuronal cell counts and biochemical assays were performed to further analyze outcomes.

**Results:**

All groups exhibited normal locomotor activity. The scopolamine group displayed longer escape latency times, reduced time in the target quadrant, decreased number of surviving neurons, and increased malondialdehyde and decreased glutathione levels compared with the control group. However, pre-treatment with 1000 mg/kg PFE notably mitigated the neurotoxic effects of scopolamine.

**Conclusion:**

The neuroprotective properties of PFE are highlighted, suggesting its potential as a promising treatment strategy for AD.

## Introduction

Alzheimer’s disease (AD) is a progressive neurological condition that primarily affects memory and cognitive function in older adults. Globally, approximately 50 million individuals suffer from dementia, with AD being the leading cause, accounting for 60%–70% of cases [1]. The hallmark pathology of AD involves abnormal protein accumulation in the brain, specifically extracellular amyloid deposition and intraneuronal neurofibrillary tangles (NFTs) formed from hyperphosphorylated microtubule-associated tau protein. These changes contribute to neural cell death and cognitive decline. Furthermore, oxidative stress and inflammation, identified as primary contributors, have been strongly associated with the pathogenesis of AD, significantly contributing to its harmful effects [2, 3]. Donepezil, an acetylcholinesterase inhibitor, is a standard medication for treating AD symptoms. It boosts brain acetylcholine (ACh) levels and improves cognitive function in patients with AD [4]. However, approved pharmacological treatments for AD, such as rivastigmine (Exelon^®^, Novartis, Switzerland)) and galantamine (Reminyl^®^, Janssen Pharmaceuticals, Belgium), have limitations, including short half-lives and severe side effects such as diarrhea, nausea, vomiting, and hepatotoxicity [5]. Consequently, there is growing interest in alternative therapies that use safe phytochemicals from medicinal plants and foods to prevent and treat AD.

Scopolamine is commonly used to mimic AD in rodents [6–9]. An antagonist of the muscarinic ACh receptor disrupts learning and memory functions. Scopolamine competitively binds to ACh receptors located in the cholinergic regions of the brain [9]. ACh serves as a pivotal neurotransmitter in the central nervous system and plays a significant role in memory formation. The failure of ACh to bind to its receptors disrupts the signaling cascade essential for memory formation, leading to significant cognitive function deficits [10]. Scopolamine has been shown to induce various detrimental effects associated with neurotoxicity, including activation of acetylcholinesterase activity, increased lipid peroxidation, and suppression of the antioxidant defense system in the whole brain, particularly in the cortex and hippocampus, causing cognitive impairment in rat models [4, 6, 7, 9, 11]. Moreover, scopolamine induces the accumulation of amyloid precursor protein and tau in the rat cortex [12] and reduces neuronal density in the hippocampus [13]. These adverse changes are similar to those observed in AD models. It is also used to develop AD models. Therefore, choosing scopolamine as an experiment in this study offers a comprehensive and well-established model to explore the mechanisms underlying cognitive deficits. *Paederia foetida* (PF), abundantly found in North-east India and used in Thai cuisine, has been studied for its diverse phytochemical compounds such as iridoid glycoside, sitosterol, alkaloids, and volatile oils [14–16]. Numerous studies have highlighted the properties of PF leaf extract (PFE), reporting the presence of compounds like Vitamin C and various flavonoid phenolics [15, 17–19]. These findings will help us understand the rich phytochemical composition of PF leaves and their potential beneficial properties. Several studies have demonstrated the preventive effects of PFE on the decrease in reactive oxygen species (ROS), elevation of glutathione (GSH) levels, and augmentation of vital antioxidant enzyme activities, such as superoxide dismutase (SOD), catalase, glutathione (GSH), and glutathione peroxidase (GPx), in pathological rat models [14, 20, 21].

Moreover, previous studies have highlighted the therapeutic potential of PF leaves in various health aspects, including its hepatoprotective, antidiarrheal, anti-inflammatory, antihyperlipidemic, and anti-hyperglycemic effects [14, 20–23]. Oxidative stress, which is characterized by high ROS levels and low antioxidant levels, is a cause of AD [24]. Therefore, the preventive effects of PFE, particularly its antioxidant activity, might protect neurons in a scopolamine-induced rat model.

PFE was used to assess its neuroprotective effects in a rat model of scopolamine-induced cognitive impairment. The aim of this study was to investigate whether PFE could mitigate cognitive deficits caused by scopolamine administration.

## Materials and Methods

### Ethical approval

The study was approved by the Animal Ethics Committee of Walailak University (Protocol Number: WU-ACUC-65026).

### Study period and location

This study was conducted from February 2022 to April 2024, utilizing PF extract for research in Nakhon Si Thammarat Province, Thailand. The research was carried out using scientific equipment and instruments at Walailak University, Thailand.

### PF Linn. leaf extraction

PF leaves were obtained from Pak Phanang District, Nakhon Si Thammarat Province. The plant species were identified by Dr. Prateep Panyadee from Queen Sirikit Botanic Garden, The Botanical Garden Organization, Chiang Mai, Thailand (QBG No. 133718). PF leaves were collected from February to May 2022.

PF leaves were separated from other parts of the plant and gently washed with tap water. After rinsing, the leaves were dried at 40°C for 72 h in a hot air oven until fully dried and then ground into a coarse powder. Fifty grams of the PF powder were treated with 500 mL of 95% ethanol for 7 days at room temperature (25°C). The powder was separated from the filtrate using Whatman filter paper (No. 1). The concentrated filtrate was obtained using a rotary vacuum evaporator under reduced pressure at 40°C (Heidolph Hei-VAP Advantage Rotary Evaporator, Heidolph Instruments GmbH & Co. KG, Germany). The concentrated extract was dried at 40°C in a water bath, resulting in a crude semisolid residue with a percentage yield of 30%. The final yield was stored at 4°C until further investigation. The crude extract of PF leaves was used in animal studies by suspending it in a 0.25% solution of carboxymethyl cellulose (CMC) in distilled water.

### Liquid chromatography-mass spectrometer (LC-MS) analysis

LC-MS (Agilent Technologies, USA) was employed to analyze the compound composition within PFE. Specifically, LC-MS/MS in negative ion mode was conducted using a TOF/Q-TOF mass spectrometer (6200 series TOF/6500 series Q-TOF, Agilent, US). This method enabled the comprehensive identification and characterization of compounds present in the extracts. PFE was dissolved in 20 mg/mL of 95% ethanol. The solution was filtered through a 0.2-mm membrane before injection. A 2-μl sample was injected into a UHPLC column (Zorbax Eclipse Plus C18 Rapid Resolution HD, 150 mm length × 2.1 mm in inner diameter, and particle size 1.8 μm (Agilent) at 30°C. The gradient mobile phase was a mixture of solvent A: water (LC-MS grade) with 0.1% formic acid and solvent B: acetonitrile (LC-MS grade) at a flow rate of 0.2 mL/min. The mass spectra were obtained over the m/z range of 50–1,100 m/z. The reference masses for negative electrospray ionization were set to 112.9856 and 1033.9881 m/z.

### Animals

Male rats were included in this study to avoid the influence of sex on learning and memory outcomes [25]. Male Wistar rats with a mean body weight of 150–180 g were purchased from Nomura Siam International, Thailand. They were provided with commercial food and sterile distilled water and housed in well-ventilated cages with a regular light/dark cycle (12/12 h), constant room temperature (23°C ± 2°C), and relative humidity (50%–60%). The rats had free access to a standard diet and water throughout the experimental period. The animals were acclimatized for 7 days before starting the experiments. Body weight was recorded every 3 days throughout the experiments.

### Chemicals

Scopolamine hydrobromide and donepezil were purchased from Sigma-Aldrich Chemical Co. (St Louis, MO, USA). Scopolamine and donepezil were dissolved in sterile water, whereas PFE was dissolved in 0.25% CMC and sterile water.

### Experimental design

Forty-two healthy male Wistar rats were divided into six groups, each containing seven animals (n = 7), as follows:

Group 1 (Control) received 0.25% CMC p. o. + Sterile water i. p.

Group 2 (Scopolamine group) received 0.25% CMC p. o. + Scopolamine 0.7 mg/kg i. p.

Group 3 (Low dose group) received 250 mg/kg PFE p. o. + Scopolamine 0.7 mg/kg i. p.

Group 4 (Medium dose group) received 500 mg/kg PFE p. o. + Scopolamine 0.7 mg/kg i. p.

Group 5 (High dose group) received 1,000 mg/kg PFE p. o. + Scopolamine 0.7 mg/kg i. p.

Group 6 (Donepezil group) received 0.5 mg/kg donepezil p. o. + Scopolamine 0.7 mg/kg i. p.

Based on the previous study by Rajashri *et al*. [8], a dose of 0.7 mg/kg of scopolamine administered intraperitoneally (i. p.) is sufficient to induce neurological impairment, whereas 0.5 mg/kg of donepezil significantly attenuates the effects of scopolamine. In addition, a dose of 500 mg/kg PFE exhibits antioxidant activity [14]. Therefore, we selected this dosage for our study and included two additional dosages; one lower (250 mg/kg) and one higher (1,000 mg/kg) to cover a range of potentially protective doses against scopolamine-induced effects in rats. PFE was administered orally to rats in groups 3, 4, and 5. All rats in each group, except Group 1 (control), were intraperitoneally injected with scopolamine at a dose of 0.7 mg/kg from day 15^th^ to day 28^th^. Throughout the experimental period, rats in all groups were granted free access to a standard diet and sterile water.

**Figure-1:**
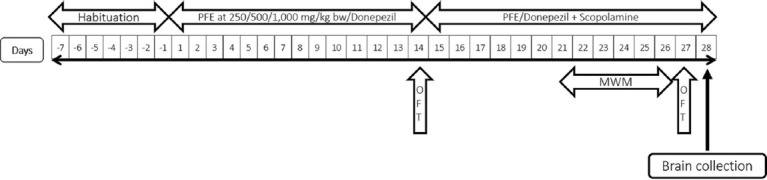
Protocol schedule to determine the neuroprotective effect of *Paederia foetida* leaf extract against scopolamine-induced memory and cognitive dysfunction. OFT=Open-field test, MWM=Morris water maze.

After a 7-day habituation period, the rats were administered PFE (250, 500, or 1000 mg/kg orally) and donepezil (0.5 mg/kg, orally) for 28 days. Specifically, PFEs were pre-treated alone for 14 days and then co-administered with scopolamine (0.7 mg/kg, i. p.) for another 14 days. The rats underwent locomotor activity (LMA) testing 2 times, which were on the 14^th^ day (before scopolamine induction) and the 27^th^ day (after 12 days of scopolamine injection). In addition, the Morris water maze (MWM) task was conducted from the 21^st^ day to the 26^th^ day, spanning 6 days. On the day following the completion of the second LMA task, rats were sacrificed for biochemical and histological studies (Figure-1).

### Behavioral studies

In this study, two behavioral tests, LMA and learning and memory performance, were employed to determine changes in behavioral performance. The open field test (OFT) was used to investigate changes in LMA and anxiety before and after scopolamine administration, whereas the MWM task was performed to investigate cognitive impairment after scopolamine administration.

### The OFT

LMA was evaluated to rule out the potential influence of drugs and interventions on locomotor function in scopolamine-treated rats. LMA was assessed using a black box with dimensions of 40 cm × 40 cm × 35 cm. On the 14^th^ day and 27^th^ day, each rat was placed at the center of the open field arena, and its LMA was recorded for 20 min using ToxTrac software (https://toxtrac.sourceforge.io, version: 2.96) [7]. Following each session, the box was cleaned with 70% alcohol and allowed to dry before placing the next rat. ToxTrac software automatically recorded the total distance traveled (cm), average speed (cm/s), and number of frozen events [26].

### MWM task

The MWM is a well-established task used to evaluate spatial learning and memory that involves hippocampus-dependent learning [27]. Rats were trained in a circular pool measuring 180 cm in diameter and 60 cm in depth with a hidden platform placed 2 cm below the water level to assess escape latency time. The pool was divided into four equal quadrants, with one quadrant used as the starting point for all trials. The pool was kept in a dark room and filled with water to a depth of 40 cm. From the 21^st^ to the 25^th^ day, rats were trained to locate the hidden platform, and the time taken to assess the learning index was recorded. Rats were allowed to swim for 2 min to find the platform; if they failed, they were guided onto the platform and allowed to stay there for 20 s. The escape latency time is the time taken by a rat to locate and escape onto the hidden platform placed in a specific quadrant of the water pool after being placed in the water at its starting location. This measurement is used as an indicator of learning and memory abilities, where shorter latency times indicate better performance in learning and memory tasks. On the 26^th^ day, the platform was removed, and the rats were allowed to explore the pool for 90 s. The time each rat spent in the target quadrant was recorded, which served as an index of spatial memory retention [4]. The target quadrant indicates the area in which the platform was positioned in the initial learning stage of the MWM test. Extended time spent in the target quadrant during the probe trial indicates enhanced spatial memory retention and an improved ability to remember the location of the platform.

### Brain sample collection

On the day following the completion of the 28-day treatment period, the rats were deeply anesthetized with 3% isoflurane. Subsequently, the brain was removed from the skull and divided into two hemispheres. The right hemisphere was fixed in 10% (v/v) formalin to determine the number of neural cells in the CA1 area of the hippocampus. The left hemisphere was rinsed with normal saline, weighed, and subsequently homogenized using 0.1 M phosphate buffer (pH 7.4) in a tissue homogenizer (IKA^®^ T10 Basic ULTRA-TURRAX® Homogenizer System, Guangzhou, China). The buffer was added at a 1:10 (w/v) ratio and centrifuged at 9503× *g* for 10 min in a refrigerated centrifuge (Thermo Scientific Heraeus® Biofuge® Stratos, USA). The supernatant was stored at –70°C until malondialdehyde (MDA) and GSH levels were determined.

### Estimation of protein concentrations

The Bradford method was used to estimate the protein content in brain tissue [8]. A 20-μL aliquot of each brain homogenate was mixed with 200 μL of Bradford reagent and incubated at 37°C for 15 min. The absorbance was measured at 596 nm using a microplate spectrophotometer system (EONC, Biotek, South Korea). Bovine serum albumin was used as the standard concentration in the range of 0.1–1 mg. The protein concentration in the brain homogenate was expressed as mg/mL [8].

### Estimation of MDA levels

The quantity of MDA, an end-product of lipid peroxidation known to harm brain tissue, was determined using the following procedure: 20 μL of brain homogenate was mixed with 20 μL of 8.1% (w/v) sodium dodecyl sulfate (SDS), 150 μL of 20% (v/v) acetic acid (pH adjusted to 3.5), and 150 μL of 0.8% (w/v) TBA in an aqueous solution. The total volume was adjusted to 400 μL with distilled water and incubated at 95°C in a water bath for 60 min. After cooling, 1 mL of distilled water and 5 mL of an n-butanol/pyridine mixture (in a ratio of 15:1 v/v) were added to the mixture. The resulting mixture was centrifuged at 1,517× *g* for 10 min, and the organic layer was measured at 532 nm. The MDA concentration was calculated and expressed as μmol/mg of protein [8, 28].

### Estimation of reduced glutathione (GSH) levels

GSH, a crucial component of the human antioxidant system, was assessed using a reaction mixture composed of 10 μL of brain homogenate, 220 μL of 0.25 M sodium phosphate buffer (pH 7.4), and 126 μL of 0.04% 5,5-dithiobis (2-nitrobenzoate). The total volume was adjusted to 300 μL using distilled water, and the absorbance was measured at 412 nm. The findings were quantified and expressed as μmol/mg protein [8, 29].

### Histopathology of the brain

The right brain samples were immersed in 10% neutral formaldehyde buffer for 7 days. Subsequently, the brain samples were rinsed 3 times with PBS (pH 7.4) and then subjected to tissue processing and paraffin embedding. Using a microtome, brain samples were sectioned to obtain 5 μm slices. These sections were stained with hematoxylin and eosin (H&E) and observed under a bright-field microscope (Olympus DP27 color camera, Olympus Corporation, Japan) at 200× magnification. The quantification of surviving neurons in the CA1 area of the hippocampus was analyzed independently with a blinded approach by three researchers and reported as the number of neurons in 402 mm^2^.

### Statistical analysis

The data are presented as mean ± standard error of the mean. The paired-sample t-test was used to analyze LMA data. Parameters such as escape latency and time spent in the target quadrant during the MWM task and biochemical assays were assessed using one-way analysis of variance, followed by *post hoc* tests utilizing the least significant difference test. Statistical significance was considered for p-values less than 0.05.

## Results

### Characterization of constituents in PFE using liquid chromatography-mass spectrometer (LC-MS/MS)

**Figure-2:**
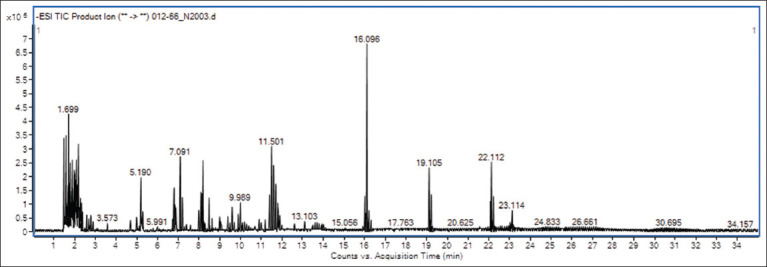
Negative ionization mode chromatogram of *Paederia foetida* leaf extract by liquid chromatography-mass spectrometer/MS.

PFE was subjected to qualitative analysis using LC-MS/MS in the negative ionization mode, facilitating the identification of multiple bioactive compound constituents (Figure-2). The chromatographic analysis delineated a distinct peak within the retention time range of 2–35 min, indicating the elution of numerous constituents of PFE. Specifically, the analysis revealed the presence of notable flavonoid compounds in PFE, including quercetin 3-galactoside and kaempferol. Furthermore, the examination identified several phenolic compounds in the extract, such as quinic acid, chlorogenic acid, hieracian, 5-(3’,5’-Dihydroxyphenyl)-gamma-valerolactone, and m-salicylic acid (Table-1). These findings underscore the diverse array of flavonoids and phenolic compounds present in PFE. Each identified compound has potential implications for the extract’s biological activity and therapeutic significance.

**Table-1:** Identification of flavonoid and phenolic compounds in *Paederia foetida* leaf extract by liquid chromatography-mass spectrometer/MS.

No.	RT	Mass (g/mol)	Formula	Identified compounds
Flavonoid compounds				
1	2.020	464.0987	C_21_H_20_O_12_	Quercetin3-galactoside
2	2.785	286.0482	C_15_H_10_O_6_	Kaempferol
Phenolic compounds				
1	1.832	192.064	C_7_H_12_O_6_	Quinic acid
2	1.832	354.096	C_16_H_18_O_9_	Chlorogenic acid
3	2.408	302.043	C_15_H_10_O_7_	Hieracin
4	2.709	208.073	C_11_H_12_O_4_	5-(3’,5’- Dihydroxyphenyl)- gamma-valerolactone
5	2.747	138.031	C_7_H_6_O_3_	m-Salicylic acid

RT=Retention time, g=gram

### Effect of PFE on LMA in an OFT

**Figure-3:**
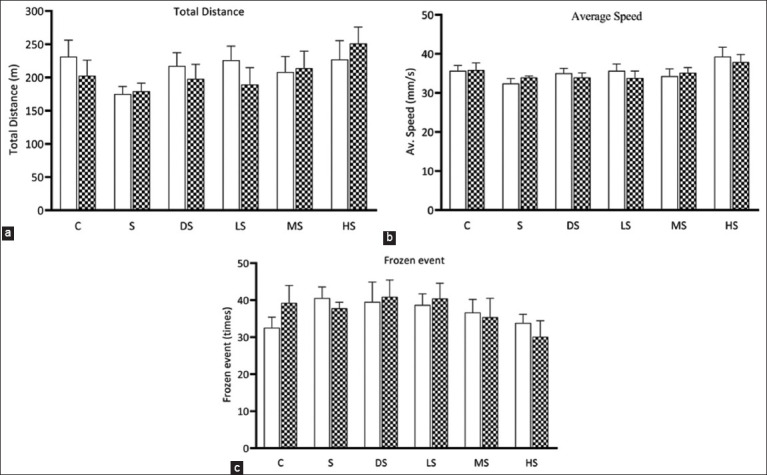
Locomotor activity in the open-field test. The white and checkered columns represent locomotor activity before and after scopolamine administration, respectively. Total distance, b: Average speed, c: Frozen event. C: Normal control; S: Scopolamine group; DS: Donepezil + scopolamine; LS: PFE 250 mg/kg + scopolamine; MS: PFE 500 mg/kg + scopolamine.; HS: PFE 1000 mg/kg + scopolamine. PFE=*Paederia foetida* leaf extract.

The OFT was used to assess changes in LMA before and after scopolamine administration to rule out any drug-induced interference with LMA and anxiety [7]. The total distance traveled, average speed, and number of freezing events in the open field arena were recorded using ToxTrac software. The results showed no significant differences in the total distance traveled, average speed (mm/s), or number of freezing events among the six groups before and after scopolamine administration (p > 0.05) (Figure-3). These findings indicated that scopolamine administration at a dose of 0.7 mg/kg i. p. for 14 days did not affect LMA or induce anxiety in the rats used in this study.

### Effects of PFE on learning and memory in the MWM task

**Figure-4:**
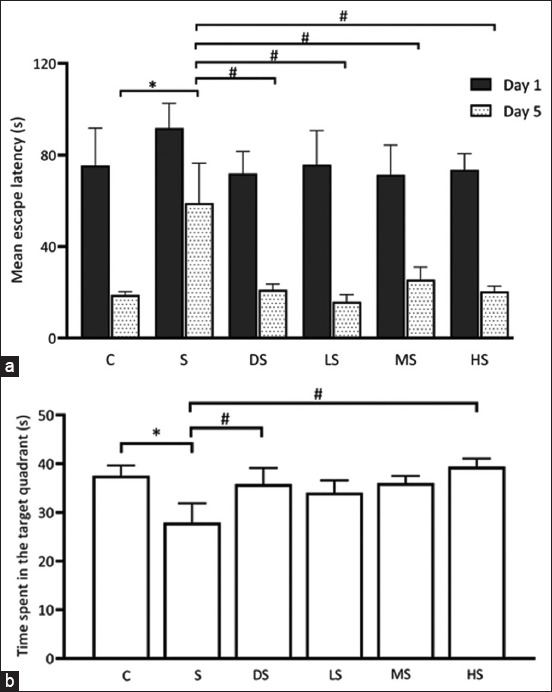
Morris water maze task. The graph shows the mean escape latencies during the acquisition task on day 1 and day 5 (a). Bar graphs illustrate the mean time taken to reach the target quadrant during the probe trial on day 6 (b). * p < 0.05 versus C; # p < 0.05 versus S. C: Normal control; S: Scopolamine group; DS: Donepezil + scopolamine; LS: PFE 250 mg/kg + scopolamine; MS: PFE 500 mg/kg + scopolamine.; HS: PFE 1000 mg/kg + scopolamine. PFE=*Paederia foetida* leaf extract.

The assessment of spatial learning and memory in rats, which are crucially dependent on hippocampal function, was conducted using an MWM task. The mean escape latency time (mELT) evaluated across days 21–25 (training day 1–5), served as an indicator of learning ability. Initially, no significant differences in mELT were observed among the six experimental groups on the first training day of the MWM task (p > 0.05). However, as training progressed, a slight decrease in mELT was evident in all groups from the 2^nd^ to the 5^th^ training days. Notably, by the 5^th^ day, the rats administered scopolamine displayed the longest mELT in the MWM task. Specifically, on the 5^th^ day, the scopolamine-treated rats exhibited significantly longer mELT than the control, donepezil, and all PFE dose (250, 500, or 1000 mg/kg) groups (p < 0.05), as shown in Table-2 and Figure-4a. These results suggest that all PFE doses potentially counteract the adverse effects of scopolamine.

**Table-2:** Mean escape latency time (second) in Morris water maze task.

Training day					

Group	Day 1	Day 2	Day 3	Day 4	Day 5
C	94.86 ± 7.43	31.57 ± 8.10	47.29 ± 10.95	18.57 ± 4.61	18.86 ± 1.49
S	91.76 ± 10.76	42.43 ± 10.51*	50.43 ± 13.89	32.57 ± 9.19*	59.00 ± 17.51*
DS	71.86 ± 9.75	45.29 ± 13.33	25.57 ± 4.77^#^	24.00 ± 5.43^#^	21.14 ± 2.48^#^
LS	64.14 ± 9.46	31.00 ± 6.313^#^	23.00 ± 6.83^#^	14.00 ± 3.31^#^	15.86 ± 3.16^#^
MS	56.19 ± 6.67	42.86 ± 12.99	20.00 ± 7.35^#^	20.43 ± 3.05^#^	25.57 ± 5.49^#^
HS	73.57 ± 7.03	37.14 ± 4.66^#^	26.57 ± 6.47^#^	15.29 ± 3.48^#^	20.43 ± 2.31^#^

Significance is indicated by

* p < 0.05 versus control group;

# p < 0.05 versus scopolamine group. C=Normal control; S=Scopolamine group; DS=Donepezil + scopolamine; LS=250 mg/kg PEE + scopolamine; MS=500 mg/kg PPE + scopolamine.; HS=1000 mg/kg PPE + scopolamine

Following the training phase, a probe trial on the 6^th^ day aimed to assess retention memory in the MWM. Spatial memory retention was quantified by calculating the mean time spent in the target quadrant. The results of the probe trial indicated that the mean times spent in the target quadrant for the control, scopolamine, donepezil, and low, medium, and high doses of PFE groups were 37.57 ± 2.08, 27.93 ± 3.94, 35.86 ± 3.28, 34.07 ± 2.51, 36.07 ± 1.43, and 39.43 ± 1.64 s, respectively (Figure-4b). Notably, a significantly lower mean time spent in the target quadrant was observed in the scopolamine group than in the control group (p < 0.05). In addition, significant differences were evident between the scopolamine and donepezil groups and between the low- and high-dose PFE groups (p < 0.05). Collectively, these findings indicate the potential of PFE to improve scopolamine-induced memory impairment in rats.

### Effects of PFE on MDA levels

**Figure-5:**
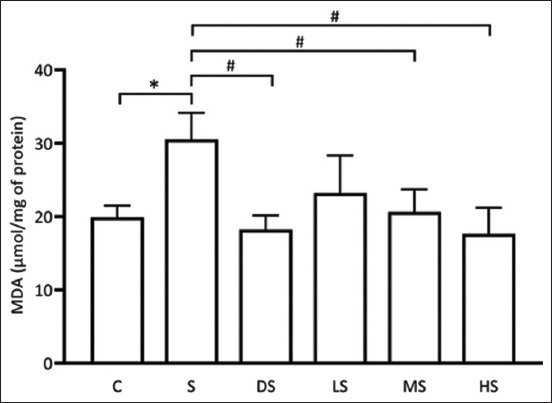
Effects of *Paederia foetida* leaf extract on MDA levels. * p < 0.05 versus C; # p < 0.05 versus S. C: Normal control; S: Scopolamine group; DS: Donepezil + scopolamine; LS: 250 mg/kg PPE + scopolamine; MS: 500 mg/kg PPE + scopolamine.; HS: 1000 mg/kg PPE + scopolamine.

MDA is a by-product of lipid peroxidation, particularly in the brain, where lipids are a predominant structural component [30]. Elevated levels of ROS in the brain lead to increased MDA production due to enhanced lipid peroxidation. Increased MDA levels serve as a marker of oxidative stress in brain tissue. In this study, the assessment of MDA levels revealed a significant increase in the scopolamine-induced group compared with the control group (p < 0.05). However, both the PFE- and donepezil-treated groups exhibited noteworthy reductions in MDA levels, demonstrating a significant decrease relative to the scopolamine-treated group (p < 0.05*)*, as depicted in Figure-5. These findings strongly suggest that PFE may effectively mitigate ROS generation in scopolamine-treated rats, as indicated by reduced MDA levels.

### Effects of PFE on reduced Glutathione (GSH) levels

**Figure-6:**
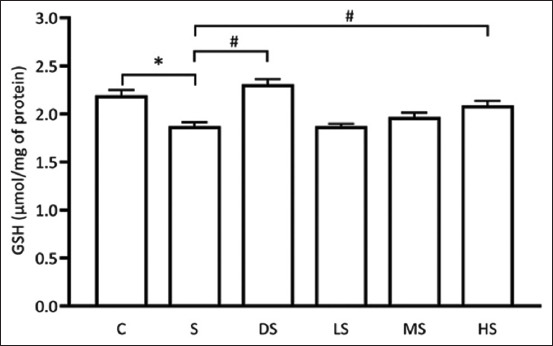
Effects of *Paederia foetida* leaf extract on GSH levels. *p < 0.05 versus C; #p < 0.05 versus S. C: Normal control; S: Scopolamine group; DS: Donepezil + scopolamine; LS: 250 mg/kg PPE + scopolamine; MS: 500 mg/kg PPE + scopolamine.; HS: 1000 mg/kg PPE + scopolamine.

GSH functions as a crucial substrate for GPx in the reaction that neutralizes hydrogen peroxide, transforming these harmful ROS into water and alleviating oxidative stress in cells [31]. Adequate GSH levels protect the brain from oxidative damage. This study revealed a significant decrease in GSH levels in the scopolamine-induced group compared with the control group. However, both the PFE- and donepezil-treated groups exhibited a remarkable elevation in GSH levels, demonstrating a significant increase compared with the scopolamine-treated group (p < 0.05), as illustrated in Figure-6. These findings strongly suggest that PFE may effectively counteract ROS generation in scopolamine-treated rats, as evidenced by the elevated GSH levels. This action parallels the effects observed in the donepezil group, implying a potential anti-oxidative mechanism of PFE in mitigating ROS production induced by scopolamine.

### Effect of PFE on the survival of pyramidal hippocampal neurons

**Figure-7:**
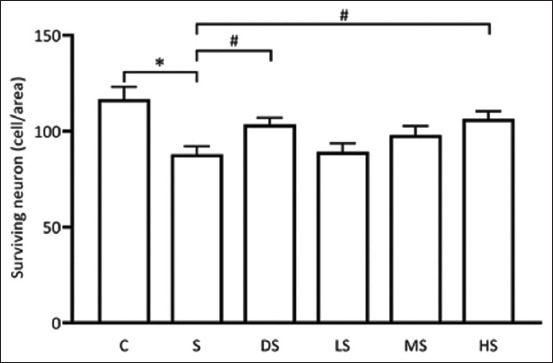
Bar graph to illustrate the number of surviving pyramidal neurons in the CA1 area. *p *<* 0.05 versus C; #p *<* 0.05 versus S. C: Normal control; S: Scopolamine group; DS: Donepezil + scopolamine; LS: 250 mg/kg PPE + scopolamine; MS: 500 mg/kg PPE + scopolamine.; HS: 1000 mg/kg PPE + scopolamine.

**Figure-8:**
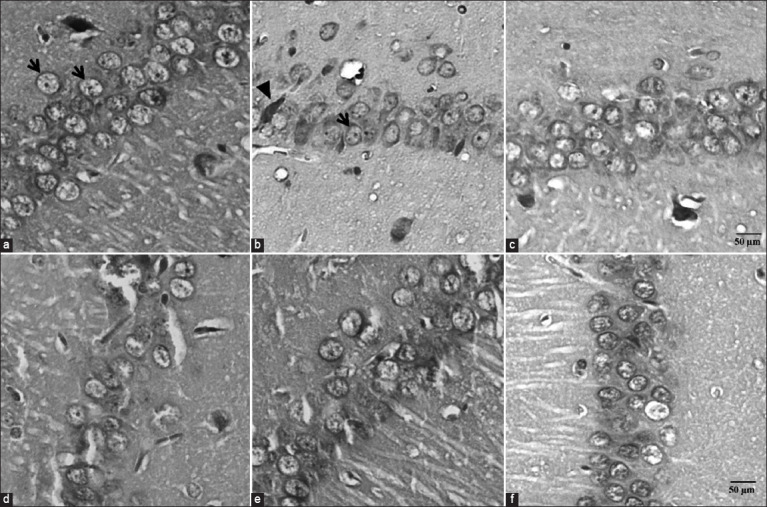
Effect of PFE on pyramidal neurons in the hippocampus. (a) Normal control, (b) Scopolamine group, (c) Donepezil + scopolamine, (d) PFE 250 mg/kg + scopolamine, (e) PFE 500 mg/kg + scopolamine., (f) PFE 1,000 mg/kg + scopolamine. Survival and dead cells are indicated by arrows and heads, respectively. The images were captured at 200× magnification. PFE=*Paederia foetida* leaf extract.

The current study aimed to elucidate the neuroprotective potential of PFE by assessing the number of surviving pyramidal neurons in the CA1 region of the hippocampus. Brain tissue samples were acquired and subjected to H&E staining. The results revealed significant differences in the numbers of surviving neurons among the experimental groups. Notably, the results exhibited that the number of surviving neurons was significantly lower in the scopolamine-induced group (87.99 ± 4.13) than in the control group (116.76 ± 6.39), p *<* 0.05. Conversely, the donepezil-treated group exhibited a significantly higher number of surviving neurons (103.57 ± 3.44) than the scopolamine-induced group, p < 0.05. Furthermore, the group treated with high-dose PFE exhibited a significantly higher count compared with the scopolamine-induced group, with a significance level of p < 0.05 (Figures-7 and 8). These findings indicated that high-dose PFE exerted a protective effect on hippocampal cells in scopolamine-induced rats, suggesting its potential neuroprotective effects.

## Discussion

AD is a devastating neurodegenerative disorder that causes cognitive impairment and memory loss. The characteristic features of AD include the accumulation of amyloid-beta (Aβ) plaques and NFTs in the brain, which are believed to contribute to neuronal dysfunction and damage [32, 33]. Oxidative stress is a significant factor in the pathophysiology of AD, instigating an imbalance between free radicals and the antioxidant system. Oxidative stress has been demonstrated in rodent AD models and is considered pivotal to disease development and progression [3, 24, 34]. Cellular structures, cell membranes, proteins, and nucleic acids are notably susceptible to ROS. The deterioration of these structures leads to cell death and impairment of specific cellular functions. The whole brain, rich in polyunsaturated fatty acids, is a prime target for ROS-induced lipid peroxidation [30]. This process triggers neuronal cell death, ultimately contributing to cognitive impairment [35].

Consequently, the brain is susceptible to scopolamine-induced lipid peroxidation through free radical reactions, leading to elevated MDA levels, a marker of lipid peroxidation [4, 8, 36]. Our findings are consistent with those of previous studies, demonstrating an increase in MDA levels in the scopolamine-treated group. This consistency further supports and validates our findings. Although the brain has a high metabolic rate, its relatively low levels of antioxidants make it susceptible to oxidative stress [37]. Glutathione (GSH), a non-enzymatic antioxidant system comprising glutamic acid, cysteine, and glycine, plays a pivotal role as the first line of defense against oxidative stress. The role of GSH is to protect cells against oxidative stress by neutralizing free radicals directly or indirectly through enzymatic reactions. GSH activates GPx, converting harmful H_2_O_2_ into nontoxic forms, thus mitigating oxidative stress in the brain [38]. This study revealed that scopolamine induces an increase in MDA levels and a decrease in GSH levels in brain tissue, which is concordant with prior research [4, 8, 36] and supports our findings.

The behavioral and histological assessments conducted on the scopolamine-treated rats in this study revealed significant insights. Initially, the administration of scopolamine did not significantly affect the LMA of the rats, suggesting no notable impact on their overall motor function. A previous study by Jafarian *et al*. [7] showed that a single dose of 20 mg/kg scopolamine-induced hyperactivity and anxiety-like behaviors in rats within 1 h. However, our study used a lower dose of scopolamine (0.7 mg/kg daily for 14 days) to gradually induce memory impairment, aiming to mimic AD in aging humans. The lower dose may explain why LMA remained intact. This discrepancy may be attributable to the different doses used by Jafarian *et al*. [7]. The MWM task, renowned for evaluating spatial learning and memory in rodents, particularly involves hippocampal-dependent learning [39]. The hippocampus plays a critical role in diverse cognitive functions and is primarily recognized for its involvement in memory formation and spatial navigation. It is also responsible for the conversion of short-term memories into long-term memories, which is an essential process in learning and memory consolidation [40]. This brain region consists of several subfields, such as the dentate gyrus, CA1, CA2, CA3, and subiculum, each contributing uniquely to memory processing [41]. The pyramidal neurons in the CA1 region of the hippocampus are notably susceptible to various adverse conditions such as oxidative stress, ischemia, hypoxia, inflammation, and excitotoxicity [42–44]. Scopolamine-induced oxidative stress damages pyramidal neuronal cells in the hippocampus, triggering apoptosis, and cell death and resulting in memory deficit. Evidence suggests that scopolamine induces neural damage in the hippocampus by decreasing the number of surviving neurons and consequent impairment of learning and memory processes [8]. In this study, rats subjected to daily scopolamine administration for 14 consecutive days displayed remarkable deficiencies in learning and memory. This was evidenced by prolonged escape latency during the acquisition trial, reflecting impaired spatial learning and reduced time spent in the target quadrant during memory retrieval assessment in the scopolamine-induced group. These findings are consistent with prior studies reporting scopolamine-induced cognitive impairment in various behavioral tasks, including the elevated plus maze, shuttle box, and novel object recognition tests [4, 8, 36].

This study delineated the neuroprotective potential of PFE against scopolamine-induced harmful effects on rat brains and behavioral performance. Our findings revealed that the administration of PFE for 14 consecutive days before scopolamine treatment initiated multifaceted protective mechanisms. This intervention prevented the increase in MDA levels, halted the decrease in GSH levels, preserved pyramidal neurons in the hippocampus, and mitigated the cognitive deficits induced by scopolamine observed in the MWM task. These findings suggested that PFE possesses antioxidant activity against excessive ROS production induced by scopolamine. LC-MS/MS analysis revealed numerous phenolic compounds in PFE, such as chlorogenic acid, hieracin 5-(3′,5′-dihydroxyphenyl)-gamma-valerolactone, and salicylic acid. Flavonoid compounds, including quercetin and kaempferol, were also identified. Previous studies have reported the neuroprotective effects of these individual compounds, thereby underscoring their neuroprotective efficacy. Chlorogenic acid, quercetin, and kaempferol are known for their neuroprotective effects, largely due to their strong antioxidant properties. Kwon [45] demonstrated that chlorogenic acid mitigates scopolamine-induced cognitive impairment in rats, as measured by the MWM and Y-maze tasks.

In addition, chlorogenic acid reduced MDA levels in the hippocampus and cortex [45]. Quercetin and kaempferol are also known for their potent anti-oxidant activity against AD [46]. Pattanashetti *et al*. [47] presented evidence supporting quercetin’s ability to attenuate learning and memory impairment, increase GSH, decrease MDA, and protect neuron cells in the hippocampus of scopolamine-induced rats. These compounds exhibit the potential to improve cognitive impairment and reduce oxidative stress in diverse animal models. The presence of these compounds in plant foods and our extract (PFE) suggests potential of dietary interventions to address cognitive decline and neurodegenerative conditions, such as AD. Furthermore, studies have identified various active compounds in PFE, such as iridoid glycosides (asperuloside, scandoside, and paederoside), volatile oils (linalool, geraniol, and α-terpineal), triterpenoid (ursolic acid and oleanolic acid), β-sitosterol, arachidic acid, flavonoids, and substantial proportions of mineral elements, which are implicated in diverse bioactivities [15].

In our study, PFE administered at a dose of 1000 mg/kg demonstrated significant neuroprotective effects in scopolamine-induced rats. The treatment effectively prevented lipid peroxidation, preserved antioxidant activity, and protected pyramidal neurons in the hippocampus, resulting in improved learning and memory. These findings suggest that PFE, with its potent antioxidant properties derived from a diverse range of phenolic and flavonoid compounds, may offer therapeutic potential for counteracting oxidative stress-related damage in neurological conditions.

## Conclusion

The results highlight the potential neuroprotective effect of PFE against scopolamine-induced cognitive impairment. PFE, especially at a dose of 1000 mg/kg, exhibited significant antioxidant properties, preventing lipid peroxidation, preserving antioxidant activity, and safeguarding pyramidal neurons in the hippocampus, ultimately ameliorating learning and memory deficits. The collective antioxidant activity of PFE, attributed to its diverse array of phenolic and flavonoid compounds, holds promise for counteracting oxidative stress-related damage in neurological conditions. Although these findings underscore the therapeutic potential of PFE in addressing memory disorders, further investigations are warranted to elucidate its underlying mechanisms and validate its clinical applicability.

## Authors’ Contributions

SK and NP: Conceived and designed the study. SK: Conducted the animal treatments. SK, PK, SN, SL, KT, and TK: Performed the biochemical assays, data analysis, and interpretation of results. SK, NP, and TK: Drafted, reviewed and edited the manuscript. All authors have read, reviewed, and approved the final manuscript.

## References

[1] Dementia (2023). https://www.who.int/news-room/fact-sheets/detail/dementia.

[2] Khan S, Barve K.H, Kumar M.S (2020). Recent advancements in pathogenesis, diagnostics and treatment of Alzheimer's disease. Curr. Neuropharmacol.

[3] Twarowski B, Herbet M (2023). Inflammatory processes in Alzheimer's disease-pathomechanism, diagnosis and treatment:A review. Int. J. Mol. Sci.

[4] Kaur R, Parveen S, Mehan S, Khanna D, Kalra S (2015). Neuroprotective effect of ellagic acid against chronically scopolamine-induced Alzheimer's type memory and cognitive dysfunctions:Possible behavioural and biochemical evidences. Int. J. Prev. Med. Res.

[5] Marucci G, Buccioni M, Ben D.D, Lambertucci C, Volpini R, Amenta F (2021). Efficacy of acetylcholinesterase inhibitors in Alzheimer's disease. Neuropharmacology.

[6] Aksoz E, Gocmez S.S, Sahin T.D, Aksit D, Aksit H, Utkan T (2019). The protective effect of metformin in scopolamine-induced learning and memory impairment in rats. Pharmacol. Rep.

[7] Jafarian S, Ling K.H, Hassan Z, Perimal-Lewis L, Sulaiman M.R, Perimal E.K (2019). Effect of zerumbone on scopolamine-induced memory impairment and anxiety-like behaviours in rats. Alzheimers Dement (N Y).

[8] Rajashri K, Mudhol S, Serva Peddha M, Borse B.B (2020). Neuroprotective effect of spice oleoresins on memory and cognitive impairment associated with scopolamine-induced Alzheimer's disease in rats. ACS Omega.

[9] Chen W.N, Yeong K.Y (2020). Scopolamine, a toxin-induced experimental model, used for research in Alzheimer's disease. CNS Neurol. Disord. Drug Targets.

[10] Chen Z.R, Huang J.B, Yang S.L, Hong F.F (2022). Role of cholinergic signaling in Alzheimer's disease. Molecules.

[11] Lee G.Y, Lee C, Park G.H, Jang J.H (2017). Amelioration of scopolamine-induced learning and memory impairment by -pinene in c57bl/6 mice. Evid. Based Complement. Alternat. Med.

[12] Bihaqi S.W, Singh A.P, Tiwari M (2012). Supplementation of *Convolvulus pluricaulis* attenuates scopolamine-induced increased tau and amyloid precursor protein (AbetaPP) expression in rat brain. Indian J. Pharmacol.

[13] Gorgani S, Jahanshahi M, Elyasi L (2019). Taurine prevents passive avoidance memory impairment, accumulation of amyloid- plaques, and neuronal loss in the hippocampus of scopolamine-treated rats. Neurophysiology.

[14] Kumar V, Anwar F, Ahmed D, Verma A, Ahmed A, Damanhouri Z.A, Mishra V, Ramteke P.W, Bhatt P.C, Mujeeb M (2014). *Paederia foetida* Linn. Leaf extract:An antihyperlipidemic, antihyperglycaemic and antioxidant activity. BMC Complement. Altern. Med.

[15] Ojha S, Raj A, Roy A, Roy S (2018). Extraction of total phenolics, flavonoids and tannins from *Paederia foetida* l. Leaves and their relation with antioxidant activity. Pharmacogn. J.

[16] Chen Y.F, Gong W, Zhao Q.J, Liu C (2024). Antinociceptive iridoid glycosides from the aerial parts of *Paederia foetida*. J. Asian Nat. Prod. Res.

[17] Osman H, Rahim A.A, Isa N.M, Bakhir N.M (2009). Antioxidant activity and phenolic content of *Paederia foetida* and *Syzygium aqueum*. Molecules.

[18] Rosli N, Tajuddin N, Shafie S (2013). Preliminary study:Vitamin C in *Paederia foetida* leaves. Open Conf. Proc. J.

[19] Chanda S, Deb L, Tiwari R.K, Singh K, Ahmad S (2015). Gastroprotective mechanism of *Paederia foetida* Linn. (*Rubiaceae*)--a popular edible plant used by the tribal community of North-East India. BMC Complement. Altern. Med.

[20] Das S, Kanodia L, Mukherjee A, Hakim A (2013). Effect of ethanolic extract of leaves of *Paederia foetida* Linn. on acetic acid induced colitis in albino rats. Indian J. Pharmacol.

[21] Sumithra M, Chitra V, Anbu J, Suman R, Shubaranshu, Nithya S (2014). Hepatoprotective activity of leaf extract of *Paederia foetida* in experimental liver cirrhosis. Invent. Rapid Ethnopharmacol.

[22] Arunkumar R, Chowdhury A, Chowdhury B (2016). Hepatoprotective activity of *Paederia foetida*
*in vitro* and *in vivo* studies. J. Evid. Based Med. Healthc.

[23] Borgohain M.P, Chowdhury L, Ahmed S, Bolshette N, Devasani K, Das T.J, Mohapatra A, Lahkar M (2017). Renoprotective and antioxidative effects of methanolic *Paederia foetida* leaf extract on experimental diabetic nephropathy in rats. J. Ethnopharmacol.

[24] Breijyeh Z, Karaman R (2020). Comprehensive review on Alzheimer's disease:Causes and treatment. Molecules.

[25] Andreano J.M, Cahill L (2009). Sex influences on the neurobiology of learning and memory. Learn. Mem.

[26] Rodriguez A, Zhang H, Klaminder J, Brodin T, Andersson P, Andersson M (2018). ToxTrac:A fast and robust software for tracking organisms. Methods Ecol. Evol.

[27] Xie Y, Song A, Zhu Y, Jiang A, Peng W, Zhang C, Meng X (2021). Effects and mechanisms of probucol on aging-related hippocampus-dependent cognitive impairment. Biomed. Pharmacother.

[28] Reilly C.A, Aust S.D (2001). Measurement of lipid peroxidation. Curr. Protoc. Toxicol., Chapter 2:Unit 2.4, 2.4.

[29] Fukuzawa K, Tokumura A (1976). Glutathione peroxidase activity in tissues of Vitamin E-deficient mice. J. Nutr. Sci. Vitaminol. (Tokyo).

[30] Jove M, Mota-Martorell N, Obis E, Sol J, Martin-Gari M, Ferrer I, Portero-Otin M, Pamplona R (2023). Lipid adaptations against oxidative challenge in the healthy adult human brain. Antioxidants.

[31] Iskusnykh I.Y, Zakharova A.A, Pathak D (2022). Glutathione in brain disorders and aging. Molecules.

[32] Ovsepian S.V, O'Leary V.B, Zaborszky L (2016). Cholinergic mechanisms in the cerebral cortex:Beyond synaptic transmission. Neuroscientist.

[33] Kaur D, Behl T, Sehgal A, Singh S, Sharma N, Bungau S (2021). Multifaceted Alzheimer's disease:Building a roadmap for advancement of novel therapies. Neurochem. Res.

[34] Tonnies E, Trushina E (2017). Oxidative stress, synaptic dysfunction, and Alzheimer's disease. J. Alzheimers Dis.

[35] Jomova K, Vondrakova D, Lawson M, Valko M (2010). Metals, oxidative stress and neurodegenerative disorders. Mol. Cell. Biochem.

[36] Rabiei Z, Setorki M (2018). Effect of hydroalcoholic *Echium amoenum* extract on scopolamine-induced learning and memory impairment in rats. Pharm. Biol.

[37] Niedzielska E, Smaga I, Gawlik M, Moniczewski A, Stankowicz P, Pera J, Filip M (2016). Oxidative stress in neurodegenerative diseases. Mol. Neurobiol.

[38] Lushchak V.I (2012). Glutathione homeostasis and functions:Potential targets for medical interventions. J. Amino Acids.

[39] Vorhees C.V, Williams M.T (2014). Assessing spatial learning and memory in rodents. ILAR J.

[40] Hernandez-Mercado K, Zepeda A (2021). Morris water maze and contextual fear conditioning tasks to evaluate cognitive functions associated with adult hippocampal neurogenesis. Front. Neurosci.

[41] Jarrard L.E (1993). On the role of the hippocampus in learning and memory in the rat. Behav. Neural Biol.

[42] Wang X, Michaelis E.K (2010). Selective neuronal vulnerability to oxidative stress in the brain. Front. Aging Neurosci.

[43] Huang T.T, Leu D, Zou Y (2015). Oxidative stress and redox regulation on hippocampal-dependent cognitive functions. Arch. Biochem. Biophys.

[44] Hackett M.J, Hollings A, Caine S, Bewer B.E, Alaverdashvili M, Takechi R, Mamo J.C.L, Jones M.W.M, de Jonge M.D, Paterson P.G, Pickering I.J, George G.N (2019). Elemental characterisation of the pyramidal neuron layer within the rat and mouse hippocampus. Metallomics.

[45] Kwon S.H, Lee H.K, Kim J.A, Hong S.I, Kim H.C, Jo T.H, Park Y.I, Lee C.K, Kim Y.B, Lee S.Y, Jang C.G (2010). Neuroprotective effects of chlorogenic acid on scopolamine-induced amnesia via anti-acetylcholinesterase and anti-oxidative activities in mice. Eur. J. Pharmacol.

[46] Alexander C, Parsaee A, Vasefi M (2023). Polyherbal and multimodal treatments:Kaempferol- and quercetin-rich herbs alleviate symptoms of Alzheimer's disease. Biology.

[47] Pattanashetti L.A, Taranalli A.D, Parvatrao V, Malabade R.H, Kumar D (2017). Evaluation of neuroprotective effect of quercetin with donepezil in scopolamine-induced amnesia in rats. Indian J. Pharmacol.

